# Cut-Off Values of Visceral Adiposity to Predict NAFLD in Brazilian Obese Adolescents

**DOI:** 10.1155/2013/724781

**Published:** 2013-12-07

**Authors:** Ana Paula Grotti Clemente, Bárbara Dal Molin Netto, Aline di Piano Ganen, Lian Tock, Danielle Arisa Caranti, Marco Túlio de Mello, Sergio Tufik, Ana R. Dâmaso

**Affiliations:** ^1^Post Graduate Program of Nutrition, Universidade Federal de São Paulo, Escola Paulista de Medicina (UNIFESP-EPM), Rua Marselhesa, 630 Vila Clementino, 04020-060 São Paulo, SP, Brazil; ^2^Weigth Science Department, Rua Teodoro Sampaio, 744 Jardim Paulista, 05406-000 São Paulo, Brazil; ^3^Biosciences Department, Universidade Federal de São Paulo, Campus Baixada Santista, Rua Silva Jardim 136, 11015-020 Santos, Brazil; ^4^Department of Psychobiology, Universidade Federal de São Paulo, Escola Paulista de Medicina (UNIFESP-EPM), Rua Marselhesa, 630 Vila Clementino, 04020-060 São Paulo, SP, Brazil; ^5^Post Graduate Program of Interdisciplinary Health Sciences, Universidade Federal de São Paulo, Campus Baixada Santista, Rua Silva Jardim 136, 11015-020 Santos, Brazil

## Abstract

*Objectives.* The present study aimed at determining cut-off points of visceral fat to predict NAFLD and analyzed metabolic disorders of obese adolescents. *Methods.* Cross-sectional study involved 165 obese adolescents ranged in age from 15 to 19 years. Glycemia, hepatic transaminases, lipid profile, and insulin resistance were analyzed. Visceral and subcutaneous fat were measured by ultrasound and body composition by plesthysmography. *Results.* The NAFLD adolescents had significantly higher values for body mass, BMI-for-age, BMI, total fat, waist circumference, and visceral fat when compared with non-NAFLD obese adolescents in both genders. Moreover, there were significant positive correlations between visceral fat with the variables BMI-for-age (*r* = 0.325,), TG (*r* = 0.277), AST (*r* = 0.509), ALT (*r* = 0.519), WC (*r* = 0.390), and visceral/subcutaneous ratio (*r* = 0.790) for NAFLD group. Total fat, triglycerides, and visceral fat were the independent predictors to NAFLD. Analysis of the ROC curves revealed cut-off points of visceral fat of 4.47 cm for girls and 4.21 cm for boys. *Conclusions.* The results may suggest that abdominal ultrasonography procedure may be a safe alternative method of assessing visceral adiposity aiming to be considered to the development of preventive and treatment strategies in obese individuals. This clinial trial is registered with ClinicalTrial.gov (NCT01358773).

## 1. Introduction

Childhood obesity is a worldwide pandemic, with consequences that include an increased incidence of metabolic disorders, which may arise in part due to visceral obesity [1]. Nonalcoholic fatty liver disease (NAFLD) is an emerging public health concern that parallels rise in obesity and metabolic syndrome (MS) [2].

There is a growing prevalence about NAFLD in obese children. Previous study with obese adolescents, specifically, found a prevalence of NAFLD affect 52% of subjects. The evidence suggests that presence of insulin resistance and visceral obesity is strongly linked to pathogenesis of NAFLD which should also be considered part of the metabolic syndrome [2, 3].

In this way, several authors suggested that NAFLD is a hepatic manifestation of MS [4]. NAFLD comprises a disease spectrum ranging from simple steatosis to steatohepatitis (NASH), with varying degrees of inflammation and fibrosis, progressing to end-stage liver disease with cirrhosis [5, 6]. Feldstein et al. [7] reported a longitudinal study, describing a follow-up of up to 20 years, and demonstrated that with children NAFLD presented shorter long-term survival than the expected survival in the general population of the same age and gender.

Much recent progress has been suggested that visceral adiposity is more influential than body mass in predicting fatty liver disease. Accordingly, Dâmaso et al. [8] demonstrated that the group of adolescents with NAFLD presented significantly higher values of BMI, visceral and subcutaneous fat, insulin, and HOMA-IR in both genders, comparing with non-NAFLD patients. Fallo et al. [9] defined waist circumference (WC) as a predictor for NAFLD in their study including 86 hypertensive obese adults. In another recently reported study, Alp et al. [10] found that BMI, WC, and waist-to-height ratio were significantly higher in obese NAFLD children group and also an increase in waist and hip circumferences was detected as the grade of liver steatosis increase.

There has been increasing interest in the last few years in the role of visceral adipose tissue and NAFLD. In fact, studies have shown that visceral adipose tissue, originally considered a passive depot for energy storage, is able to secrete a variety of substances that regulate metabolism and inflammation, also participating in the pathogenesis of NAFLD [3, 8, 11]. A previous study from our group demonstrated that each 1 cm increase in visceral adiposity, when evaluated by abdominal ultrasonographic, was associated with a 2-fold greater risk of NAFLD in obese adolescents [8]. However, in most of studies, an optimal cut-off of visceral fat as risk factor independently correlated with liver damage in terms of both the presence of steatosis and disease progression have not yet been determine.

The assessment for the prognostic of fatty liver disease is crucial to improve the clinical practice for obese individuals, particularly in those without advanced disease, but at risk of developing NAFLD. Therefore, the aims of the present study were to determine cut-off points of visceral fat to predict NAFLD and analyze metabolic disorders of obese adolescents in both genders.

## 2. Subjects and Methods

### 2.1. Study Group

The cross-sectional study involved 165 obese adolescents ranged in age from 15 to 19 years. Obese adolescents were recruited for this research from Multidisciplinary Obesity Intervention Program Outpatient Clinic of the Federal University of São Paulo. Calculations of nutritional status according to HAZ and BMI-for-age values were performed using WHO Anthro Plus 1.0.4 software. The nutritional diagnosis was based on the BMI-for-age (BAZ) for the children aged >5 years and adolescents ≤19 years of age (Z score ≥ +2SD), according to cut-off points recognised by World Health Organization [13] and agreement of the adolescents and their families to participate in a lifestyle intervention for weight loss. Noninclusion criteria were as follows: identified genetic, metabolic, or endocrine disease; chronic alcohol consumption (≥20 g/d); presence of viral hepatic diseases; previous drug use; and other causes of liver steatosis.

### 2.2. Study Protocol

A clinical screening was performed to assess and their pubertal stage and anthropometric measures were assessed (stature, body mass, BMI, and body composition). Ultrasound (US) was performed and blood sample was collected and analysed for metabolic profile. For each subject, the procedures were scheduled for the same time of day approximately, 8:00 am after an overnight fasting, to remove any influence of diurnal variations.

All adolescents were examined by a trained physician and classified regarding gender development in accordance with the postpubertal stage based on the Tanner scale (stage five) for both boys and girls [14]. Individuals who had reached the appropriate World Health Organization cut-off points (breast-stage 2 for females and genitalia-stage 3 for males) were considered pubertal [15], whilst those who had yet to attain these stages were classified nonpubertal. None of the participants presented early or delayed puberty, although levels of testosterone, luteinizing hormone, or follicle-stimulating hormone were not determined.

### 2.3. Anthropometric Measurements and Body Composition

The body mass was measured (wearing light clothes and without shoes) by single assessment using a platform scale Filizola (Indústrias Filizola S/A, São Paulo, SP, Brazil; model PL 180), with a capacity of 180 kg and an accuracy of 100 g. The stature was assessed using a stadiometer with a precision of 0.1 cm (Sanny, São Bernardo do Campo, SP, Brazil; model ES 2030). BMI values were calculated as the quotient of body mass (kg) and the square of the stature (m). For the determination of waist circumference, subjects were placed in a standing position with the abdomen and arms relaxed alongside the body, and a flexible measuring tape (1 mm accuracy) was held horizontally at the midpoint between the bottom edge of the last rib and the iliac crest. The measurements were recorded with the tape applied firmly to the skin but without compression of tissues.

Body composition was measured by air displacement plethysmography in a BOD POD body composition system (version 1.69; Life Measurement Instruments, Concord, CA, USA).

### 2.4. Serum Analysis

Blood samples (20 mL) were collected from fasted adolescents by venous puncture and transferred, as appropriate, to heparinized and nonheparinized vials. Plasma glucose was determined with the aid of a commercial kit and a UniCell DXI 800 spectrophotometer (Beckman Coulter, Fullerton, CA, USA), while specific insulin (without C peptide) was determined using an enzyme assay and an Advia 2400/Kovalent analyzer (Siemens, São Paulo, Brazil). Serum levels of total cholesterol, triglyceride (TG), high-density lipoprotein (HDL), low-density lipoprotein (LDL), the hepatic transaminases, alanine aminotransferase (ALT), aspartate aminotransferase (AST), and *γ*-glutamyl transferase (GGT) were analyzed using a commercial kit (CELM, Barueri, Brazil). We chose serum AST and ALT levels since these liver enzymes have been reported to be better correlated with the presence of hepatic fat on imaging liver than the other biomarkers, corroborating to confirm the diagnosis of NAFLD [12, 16].

Insulin resistance was assessed by homeostasis model assessment insulin resistance index (HOMA-IR). HOMA-IR was calculated by the fasting blood glucose (FBG) and the immunoreactive insulin (I): (FBG (mg/dl) × I (mU/l))/405. QUICKI was calculated as 1/(log⁡⁡I + log⁡⁡FBG). The HOMA-IR and hepatic transaminase (ALT) data were analysed according to reference values described by Schwimmer et al. [17].

### 2.5. Hepatic Steatosis, Visceral, and Subcutaneous Adiposity Measurements

The abdominal ultrasonography procedures and the measurements of visceral and subcutaneous fat tissue and fatty liver were performed by the same physician. This physician was a specialist in imaging diagnostics. A 3.5 MHz multifrequency transducer [broad band] was used to reduce the risk of misclassification. The intraexamination coefficient of the variation for ultrasound (US) was 0.8%. US measurements of intra-abdominal (visceral) and subcutaneous fat were obtained. US-determined subcutaneous fat was defined as the distance between the skin and external face of the rectus abdominal muscle, and visceral fat was defined as the distance between the internal face of the same muscle and the anterior wall of the aorta. The cut-off points for the definition of visceral obesity by ultrasonography were based on the previous methodological descriptions made by Ribeiro-Filho et al. [18]. Steatosis evaluation was performed by abdominal ultrasonography. In the present study, the group with NAFLD presented some liver steatosis grade diagnosed by US. Clinical studies showed that serum biomarkers, especially ALT and AST levels, are sensitive in the detection of NAFLD and have also been associated with a positive diagnosis by US, an increased risk of metabolic syndrome, diabetes mellitus, and cardiovascular disease, in both children and adults [16, 18, 19].

### 2.6. Statistical Analysis

Statistical analyses were performed using PASW Statistics version 19 (SPSS Inc., Chicago, IL, USA) with the level of statistical significance set at *P* < 0.05. Mean values of the nutritional parameters (age, height, weight, HAZ, BMI, and WC) and serum analyses (total cholesterol, TG, HDL, LDL, and the ALT, AST, and GGT) of the non-NAFLD and NAFLD groups, stratified according to gender, were compared using the independent *t*-test and the assumptions of homoscedasticity verified using the Levene test.

In order to evaluate the association of the visceral fat and the concentrations serum analyses on both groups by applying the Pearson test correlation coefficients as the NAFLD did present homoscedasticity. Logistic regression analysis (forward LR method) was employed to compare individuals with NAFLD using the Wald test to determine which factors should be employed as predictor variables in the final model. Logistic regression analysis (forward LR method) was employed to compare individuals with NAFLD and non-NAFLD using the Wald test to determine which factors should be employed as predictor variables in the final model. For girls, age, visceral fat, LDL-c, and TG were defined as independent variables in the regression model. And for boys age, WC, AST, visceral fat, and insulin were defined as independent variables in the regression model.

A receiver operating characteristic (ROC) curve was constructed in order to establish visceral fat cut-off points for obese boys and girls that could be used to predict NAFLD. Sensitivity was defined as the probability of visceral fat to classify correctly those subjects presenting NAFLD (true positives), whereas specificity was defined as the probability of visceral fat to classify correctly those subjects presenting non-NAFLD (true negatives). The area under the ROC curve was employed as a global measure of the general precision of visceral fat as a predictor of NAFLD, in which an area of 1 would correspond to 100% sensitivity and 100% specificity and, thus, represent a perfect test for discriminating individuals. The shortest distance in the ROC curve was calculated using the function: $\sqrt{(1-{\text{sensitivity}}^{2})+\left(1-{\text{specificity}}^{2}\right)}$, as described by Zhu et al. [20].

## 3. Results

### 3.1. Entire Group

#### 3.1.1. Comparison between NAFLD versus Non-NAFLD Groups

The present study comprised 165 obese adolescents being 104 patients Non-NAFLD and 61 with diagnoses of NAFLD. Comparing the results of both groups, the NAFLD patients had significantly higher values for body mass, BAZ, BMI, total fat, waist circumference, and visceral fat when compared with non-NAFLD obese patients in both gender. The main results are summarized in Table 1.

Among the obese adolescents with NAFLD, it is important to note a higher and significant value of insulin, total cholesterol, TG, and VLDL cholesterol when compared with Non-NAFLD participants. Additionally, patients with NAFLD resulted in major and significant value of AST and HOMA-IR compared with Non-NAFLD obese patients (Table 2).

In addition, there were significant positive correlations between visceral fat with the variables BAZ (*r* = 0.325), TG (*r* = 0.277), AST (*r* = 0.509), ALT (*r* = 0.519), GGT (*r* = −0.010), WC (*r* = 0.390), and visceral/subcutaneous ratio (*r* = 0.790) for NAFLD group (Table 3). Significant positive correlations were also observed between visceral fat with body mass (*r* = 0.301), BAZ (*r* = 0.246), BMI (*r* = 0.235), total fat (*r* = 0.220), WC (*r* = 0.210), and visceral/subcutaneous ratio (*r* = 0.771) for Non-NAFLD group. Moreover, as shown in Table 4, in the logistic regression analysis it was found that total fat (kg), triglycerides, and visceral fat were the independent predictors to NAFLD.

### 3.2. Comparison between NAFLD versus Non-NAFLD Groups by Genders

The present study found some differences between genders according to presence or not of NAFLD. The obese boys with NAFLD presented values significantly higher in body mass, BMI, total fat (% and kg), waist circumference, visceral fat and visceral/subcutaneous ratio than non-NAFLD boys. It is important to observe that obese boys with NAFLD had significantly higher values of ALT than obese boys of non-NAFLD group (52.06 ± 34.13 versus 31.06 ± 17.54), respectively.

Specifically for girls with NAFLD, the body mass, total fat (% and kg), waist circumference, and visceral fat were significantly major in regard to values found for girls of Non-NAFLD group. Moreover, considering the girls of NAFLD group, the insulin, total cholesterol, VLDL cholesterol, triglycerides, and HOMA-IR were significantly higher than observed for obese girls of Non-NAFLD (Tables 1 and 2).

### 3.3. Comparison between Genders at the Same Group

#### 3.3.1. NAFLD Group

The anthropometrical and body composition results of boys with NAFLD demonstrated significant higher values of body weight, fat free mass (% and kg), waist circumference, visceral fat, and visceral/subcutaneous ratio. Indeed, boys with NAFLD presented significant and major serum ALT and AST concentrations compared with girls NAFLD (52.06 ± 34.13 versus 25.29 ± 8.70; 32.35 ± 15.34 versus 20.85 ± 4.29), respectively. Significant and higher values were observed in the total fat (%) and HDL cholesterol of girls with NAFLD compared to boys at the same analysed group (47.37 ± 4.68 versus 41.61 ± 6.69; 46.50 ± 8.63 versus 39.88 ± 8.51), respectively.

#### 3.3.2. Non-NAFLD Group

In non-NAFLD obese boys, there were values significantly higher of body weight, fat free mass (%), waist circumference, glucose, VLDL cholesterol, triglycerides, AST, and GGT when compared with girls at the same group. However, girls of Non-NAFLD group had a significant and major value of total fat (% and kg) and HDL cholesterol (45.22 ± 4.57 versus 36.98 ± 6.53; 40.87 ± 7.44 versus 35.94 ± 7.58; 48.65 ± 10.01 versus 42.93 ± 8.40), respectively.

### 3.4. Cut-Off Point of Visceral Fat

In the logistic regression analysis, using the diagnosis to NAFLD as a dependent variable it was found that total fat (kg) (*P* = 0.045), triglycerides (*P* = 0.044), and visceral fat (*P* = 0.014) were the independent predictors to development of NAFLD (Table 4). Thus, the risk individuals presenting NAFLD were raised by 60.6% for each additional increase in visceral fat of 1 cm.

**Figure 1:**
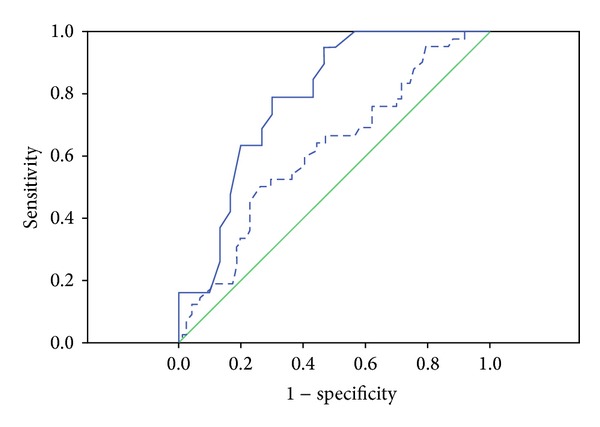
Receiver-operating characteristic (ROC) curve for the identification of NAFLD based on the visceral fat (cm) of adolescents. The area under the ROC curve of the boys was 0.784 (95% confidence interval 0.658–0.910). The WC cut-off point for the boys was 4.20 cm. And the area under the ROC curve of the girls was 0.615 (95% confidence interval 0.509–0.721). The WC cut-off point for the girls was 4.47 cm.

Moreover, analysis of the ROC curves revealed cut-off points of 4.47 cm for girls individuals and 4.21 cm for boys subjects (Figure 1). Since the area under the ROC curve for the girls group was 0.615 (*P* = 0.001) and that for boys group was 0.784 (*P* = 0.040), the difference between the respective visceral fat cut-off points was statistically significant.

## 4. Discussion

The highlighted results reinforce cut-off points of visceral fat to predict NAFLD in obese adolescents. The visceral adipose tissue, originally considered a passive depot for energy storage, is considered by previous studies as the main risk factor to development of pathogenesis of NAFLD [3, 5, 8, 11]. In our study, it was possible to determine by abdominal ultrasonography procedure a cut-off of visceral fat as risk factor for presence of NAFLD in adolescents. A noteworthy fact is that all the adolescents were obese in both NAFLD and Non-NAFLD groups. However, NAFLD adolescents presented significantly higher values for body weight, BAZ, BMI, total fat, waist circumference, visceral fat, AST and HOMA-IR, comparing with Non-NAFLD patients.

Visceral fat is strongly associated with obesity-related complications like type 2 diabetes, coronary artery disease, hypertension, and insulin resistance, which are all features of the metabolic syndrome [1, 2, 20]. The relationship between NAFLD and the features of the metabolic syndrome have been extensively reported [9, 18–22]. A study reported that 20 children diagnosed with NAFLD by abdominal ultrasonography presented increased insulin resistance and also suggested to use HOMA-IR as a screening method of NAFLD [23]. Similar results were obtained in this study that showed a higher value of HOMA-IR in NAFLD patients. Given that the liver plays a critical role in maintaining glucose and lipid homeostasis, studies have 13 demonstrated that deterioration in glucose and insulin metabolism might emerge due to the deregulation in cytokine production [increased necrosis factor alpha tumor and decreased adiponectin, among others]. Furthermore, the lipolysis leads to increases fatty acids to circulation through the liver vein portal resulting overload of beta oxidation, as well as increase ALT concentrations and accumulation of lipid in the hepatocytes [12, 24].

Moreover, elevated aminotransferases have been correlated with the presence of hepatic fat on imaging. Clinical studies showed that serum biomarkers, especially ALT levels, are sensitive in the detection of NAFLD and have also been associated with an increased risk of metabolic syndrome, diabetes mellitus, and cardiovascular disease [16, 25]. According to Devadason and Scheimann [26] unexplained alanine aminotransferase (ALT) elevation is a frequently used surrogate for the presence of NAFLD in children and adults.

In fact, we verified among the obese boys higher serum AST and ALT concentrations compared with NAFLD girls. Corroborating, another researchers found that boys had the higher prevalence of NAFLD (44%) than girls (7%) using ALT as a surrogate of NAFLD [27]. The reason for gender difference remains unclear. However, studies suggested a potential reason for a gender-based difference by estrogen-testosterone ratio as an important mediator for manifestation of insulin resistance. Sex hormones effect the distribution of both fat and muscle. Sex hormone binding globulin, produced in the liver, is strongly correlated with insulin sensitivity and higher serum ALT level [27, 28]. In addition, we verified a positive correlation between visceral fat and ALT and AST, confirming that the visceral fat can be considered a reliable and good measure to predict NAFLD once there are strong evidences in the literature that these hepatic transaminases constitute important markers of NAFLD progression [27]. Although, high serum transaminases levels should not be used alone as a parameter of NAFLD detection, considering that the expansion of visceral fat was correlated with the presence of NAFLD in a previously studies [8, 29].

In agreement, our work confirms that the visceral fat, total fat, and TG were the independent predictors to NAFLD in obese adolescents. NAFLD, in the absence of chronic alcohol consumption, has been characterized by the accumulation of large particles of TG within hepatocytes leading causes of chronic liver disease [25]. Alp et al. reported that among anthropometric and clinical parameters only total adipose tissue mass percentage measured by bioelectric impedance was positively correlated with the grades of hepatosteatosis [10]. In fact, the risk of early-onset NAFLD might be associated with a visceral adiposity rebound in adolescents linked to unbalanced diet, deregulation in the neuroendocrine energy balance and other inflammatory processes. Previous authors demonstrated that NAFLD obese subjects presented a negative correlation between adiponectinemia and neuropeptide Y (NPY)/agouti-related protein (AgRP) ratio suggested that these patients presented an inflammatory profile that can cause important influence in the neuroendocrine energy balance [30]. Indeed, in this same study, a positive correlation between visceral fat and AgRP levels in all obese patients was observed. This neuropeptide is involved in feeding behavior inducing hyperphagia. This way, the reduction of visceral fat may be considered an important strategy in the energetic balance control, reinforcing the importance of our findings, to determine an optimal cut-off of visceral fat as risk factor independently correlated liver damage.

According to the literature, the increase of visceral adipose tissue and insulin resistance are the undisputed major contributors to NAFLD [3, 5, 8, 11, 26, 30, 31]. Furthermore, the assessment of these parameters for the prognostic of fatty liver disease is crucial to improve the predictive ability of clinical procedures in obese individuals. The present study has shown the clinical importance of an abdominal ultrasonography suggestive in the pathogenesis of NAFLD. Studies have demonstrated the value of ultrasonography in the diagnosis of steatosis. Joy et al. [32] reported that ultrasound presented a high positive predictive value when suggestive of NAFLD, besides being cost-effective, safe, and patient-friendly. The results of Shannon et al. [33] indicate that hepatic ultrasonography is a useful tool for quantifying steatosis in pediatric patients who have suspected NAFLD, with ultrasonographic steatosis score strongly correlating with grade of steatosis on liver biopsy. Mottin et al. [34] found that in the ultrasonography results yielded a positive predictive value of 95.4%, indicating the validity of this test as a diagnostic tool for this comorbidity in obese patients. Ribeiro-Filho et al. [18] using ultrasound measurement observed correlation between visceral adiposity and major values of BMI when compared with Non-NAFLD obese individuals. These data corroborate with the present investigation, which demonstrated significantly higher values for body weight, BAZ, BMI, and WC in NAFLD obese adolescents.

Our findings strongly propose that visceral fat can be used as a good predictor for the possibility of liver disturbances as steatosis. Two criteria were employed in establishing visceral fat cut-off points in the study, the highest sensitivity and specificity, and the shortest distance in the ROC curve [20]. The visceral fat cut-off points, found, 4.47 cm for the girls and 4.21 cm for the boys were based on the most appropriate combination of the two criteria. The shortest distance in the ROC curve was 0.615 of girls and 0.784 of the boys. The statistical power of the adjusted model employed was excellent, as demonstrated by the high-sensitivity values obtained for the girls and boys (78.9% and 50.0%, resp.). In contrast, the specificity of the adjusted model was 74.3% for the girls and 70.0% for the boys. Another study identified cut-off point of 4.76 cm in men and 3.55 cm in women visceral fat analyzed by ultrasonography. According to the authors, in the cases of metabolic syndrome, these visceral fat cut-off values have showed to have high specificity and sensitivity for predicting the presence of cardiovascular diseases [35].

Nevertheless, we recognize limitations to the current study. The (ROC) curve of visceral fat thickness for predicting NAFLD was not great in adolescents girls. This is reflected by the shortest distance in the ROC curve of 0.615. Therefore, the visceral fat cut-off points proposed in this study can be explored with larger populations from obese adolescents in additional investigation.

In conclusion, we found that the visceral fat cut-off point for the prediction of NAFLD adolescents in both genders. Therefore, the results may suggest that abdominal ultrasonography procedure may be a safe alternative method of assessing visceral adiposity aiming to be considered to the development of preventive and treatment strategies in obese individuals. Although the visceral fat cut-off points proposed in this study cannot be generalized to other populations, the present findings constitute an important indication of the need for further studies with larger populations from different geographical/sociocultural environments.

## Figures and Tables

**Table 1:** Anthropometric and body composition characteristics of the studied population.

	Non-NAFLD						NAFLD					
	Total (*n* = 104)		Male (*n* = 30)		Female (*n* = 74)		Total (*n* = 61)		Male (*n* = 19)		Female (*n* = 42)	
	Mean	SD	Mean	SD	Mean	SD	Mean	SD	Mean	SD	Mean	SD
Age (years)	16.38	1.52	16.16	1.28	16.47	1.61	17.21	1.83^a^	16.67	1.44	17.46	1.95^b^
Weight (kg)	92.05	9.79	96.75	8.08^c^	90.14	9.82	97.05	9.04^a^	103.31	4.20^b,c^	94.21	9.24
Height (cm)	165.19	6.87	171.2	6.36^c^	162.76	5.45	165.59	7.43	171.93	5.87^a^	162.73	6.21
HAZ (Z score)	0.01	0.89	−0.13	0.95	0.08	0.86	−0.01	0.88	−0.18	0.89	0.07	0.87
BAZ (Z score)	2.76	0.44	2.78	0.42	2.76	0.45	2.95	0.44^a^	3.04	0.46^b^	2.90	0.43
BMI (kg/m^2^)	33.74	3.12	33.04	2.62	34.02	3.28	35.44	3.27^a^	35.07	3.00^b^	35.61	3.40^b^
Total fat (%)	42.84	6.39	36.98	6.53	45.22	4.57^c^	45.57	5.97^a^	41.61	6.69^b^	47.37	4.68^b,c^
Fat free mass (%)	57.16	6.42^a^	63.04	6.59^c^	54.78	4.57	54.10	6.20	57.34	7.89^b^	52.63	4.68^b,c^
Total fat (kg)	39.45	7.77	35.94	7.58	40.87	7.44^c^	44.28	7.49^a^	43.02	7.51^b^	44.86	7.50^b^
Fat free mass (kg)	52.54	7.54	60.94	6.86^c^	49.13	4.54	52.77	7.47	60.28	6.99^c^	49.38	4.72
Waist Circumference (cm)	96.34	7.47	98.49	5.45^c^	95.45	8.02	100.96	7.73^a^	105.63	6.23^b,c^	99.04	7.52^b^
Visceral fat (cm)	3.67	1.22	3.84	1.21	3.60	1.23	4.45	1.33^a^	5.24	1.39^b,c^	4.09	1.15^b,c^
Subcutaneous fat (cm)	3.53	0.87	3.41	0.81	3.58	0.89	3.54	0.73	3.47	0.63	3.57	0.78
Visceral/Subcutaneous ratio	1.09	0.43	1.18	0.41	1.06	0.43	1.32	0.49^a^	1.55	0.43^b,c^	1.21	0.48^c^

BMI: body mass index; HAZ: height-for-age Z score; BAZ: BMI-for-age.

Values are expressed as mean ± standard deviation. Mean values were significantly different according to independent *t*-test (*P* < 0.05).

^a^Comparison of the group with NAFLD versus group non-NAFLD.

^b^Comparison of the group with NAFLD versus group non-NAFLD by genders.

^c^Comparison of the genders at the same group.

**Table 2:** Biochemical characteristics of the studied population.

	Non-NAFLD						NAFLD					
	Total (*n* = 104)		Male (*n* = 30)		Female (*n* = 74)		Total (*n* = 61)		Male (*n* = 19)		Female (*n* = 42)	
	Mean	SD	Mean	SD	Mean	SD	Mean	SD	Mean	SD	Mean	SD
Glucose (mg/dL)	89.74	5.77	91.58	6.06^c^	88.89	5.48	91.8	6.52	94.29	4.92	90.56	6.92
Insulin (*μ*U/mL)	15.21	8.26	16.26	12.27	14.72	5.61	17.95	6.98^a^	17.62	6.65	18.11	7.22^b^
Total Cholesterol (mg/dL)	163.49	31.45	170.62	34.17	160.2	29.82	171.63	32.64^a^	168.12	35.42	173.38	31.57^b^
HDL-c (mg/dL)	46.84	9.85	42.93	8.40	48.65	10.01^c^	44.29	9.08	39.88	8.51	46.50	8.63^c^
LDL-c (mg/dL)	97.06	28.24	104.14	28.72	93.8	27.64	103.98	27.27	104.52	30.3	103.70	26.11
VLDL-c (mg/dL)	19.57	9.37	23.55	11.35^c^	17.75	7.75	23.43	10.13^a^	23.94	10.00	23.18	10.33^b^
TG, (mg/dL)	97.73	46.83	117.55	57.08^c^	88.62	38.47	116.67	51.05^a^	119.18	51.03	115.41	51.77^b^
AST, U/L	22.77	5.98	25.1	6.62^c^	21.7	5.38	24.69	10.84^a^	32.35	15.34^c^	20.85	4.29
ALT, U/L	27.79	14.68	31.06	17.54	26.28	13.06	34.22	24.19	52.06	34.13^b,c^	25.29	8.70
GGT, U/L	23.73	11.86	28.82	16.34^c^	21.4	8.26	29.29	22.41	37.12	33.26	25.38	13.32
HOMA-IR	3.35	1.96	3.71	2.96	3.18	1.23	4.1	1.65^a^	4.1	1.56	4.09	1.71^b^
Quick	0.32	0.02^c^	0.32	0.02	0.33	0.02	0.31	0.02	0.31	0.02	0.31	0.01^b^

Reference values: glucose (60–110 mg/dL), insulin (20 *μ*U/mL), HOMA-IR (2.0), total cholesterol (170 mg/dL), TG (33-129 mg/dL), HDL cholesterol (38 mg/dL), LDL cholesterol (<130 mg/dL), AST (10–40 U/L), ALT (10–35 U/L), and GGT (17–30 U/L) (Burgert et al. [12]).

Values are expressed as mean ± standard deviation. Mean values were significantly different according to indenpendent *t*-test (*P* < 0.05).

^a^Comparison of the group with NAFLD versus group non-NAFLD.

^b^Comparison of the group with NAFLD versus group non-NAFLD by genders.

^c^Comparison of the genders at the same group.

**Table 3:** Correlations between visceral fat, biochemical, and anthropometric parameters.

	Non-NAFLD (*n* = 104)		NAFLD (*n* = 61)	
	Pearson *R*	*P* value	Pearson *R*	*P* value
Weight (kg)	0.301	**0.002**	0.233	0.070
BAZ (Z score)	0.246	**0.014**	0.325	**0.015**
BMI (kg/m^2^)	0.235	**0.016**	0.219	0.09
Total fat (kg)	0.220	**0.025**	0.087	0.505
Fat free mass (kg)	0.143	0.147	0.192	0.138
Waist circumference (cm)	0.210	**0.031**	0.390	**0.003**
Visceral/subcutaneous ratio	0.771	**0.001**	0.790	**0.001**
VLDL-c (mg/dL)	−0.005	0.964	0.271	0.052
TG, (mg/dL)	−0.004	0.968	0.277	**0.049**
AST, U/L	−0.070	0.461	0.509	**0.001**
ALT, U/L	−0.140	0.176	0.519	**0.001**
GGT, U/L	0.016	0.271	−0.010	**0.001**

**Table 4:** Logistic regression analysis with NAFLD adolescents.

	*β*-Coefficient	*P* value
BAZ (Z score)	0.382	0.222
Total fat (kg)	1.101	**0.045**
Fat free mass (kg)	0.971	0.401
Waist circumference (cm)	1.070	0.066
Insulin (*μ*U/mL)	1.010	0.728
TG, (mg/dL)	1.009	**0.044**
AST, U/L	0.943	0.283
ALT, U/L	1.043	0.079
GGT, U/L	1.002	0.906
Visceral fat (cm)	1.606	**0.014**
